# ER Stress Induced by Artemisinin and Its Derivatives Determines the Susceptibility to Their Synergistic Apoptotic Killing With TRAIL

**DOI:** 10.1002/cam4.71001

**Published:** 2025-06-23

**Authors:** Snigdha Bhowmick, Yong J. Lee

**Affiliations:** ^1^ Department of Biomedical Sciences Cedars‐Sinai Medical Center Los Angeles California USA

**Keywords:** apoptosis, artemisinins, cytotoxicity, ER stress, ferroptosis

## Abstract

**Aim:**

Artemisinins are a class of antimalarial drugs that are lately being researched for their antitumor activity. We previously reported that artesunate, an artemisinin derivative, can induce ferroptosis and enhance TRAIL (Tumor necrosis factor‐Related Apoptosis‐Inducing Ligand)‐induced apoptosis. Here we investigated the role of endoplasmic reticulum (ER) stress induced by artemisinin and its derivatives, especially in the enhancement of TRAIL‐induced apoptosis, which can be exploited for repurposing the use of artemisinins in cancer therapy.

**Methods:**

We show in this study a comparative profile of the ER stress induced by different derivatives of this drug, namely artemisinin, artesunate, arteether, artemether, and dihydroartemisinin, all of which are available readily and approved for treating human patients. These five derivatives were used to treat human colon carcinoma HCT116 cells and pancreatic adenocarcinoma BxPC3 cells over a range of doses.

**Results:**

Our data show a highly significant positive correlation between ER stress caused by these drugs and their corresponding apoptotic susceptibilities upon treatment with TRAIL.

**Conclusion:**

We concluded that dihydroartemisinin is the most effective contender among all the derivatives tested to enhance TRAIL‐induced apoptosis.

## Graphical abstract

Endoplasmic reticulum stress response is a deterministic factor defining the death in cancer cells treated with a combination of artemisinins and TRAIL.

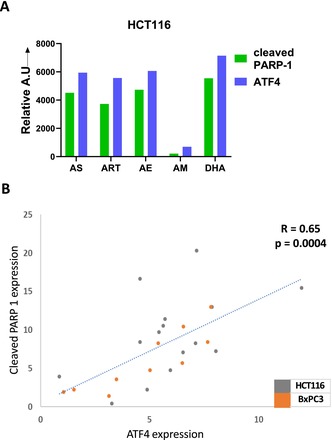

**Abbreviations**
AE: arteether
AM: artemether
ART: artemisinin
AS: artesunate
ATF4: activating transcription factor 4
BSA: bovine serum albumin
CHOP: C/EBP (CCAAT‐enhancer binding proteins) homologous protein
CYP: cytochrome P
DHA: dihydroartemisinin
ECL: enhanced chemiluminescence
ER: endoplasmic reticulum
Hb: hemoglobin
PAGE: polyacrylamide gel electrophoresis
PARP‐1: poly (ADP‐ribose) polymerase‐1
PUMA: p53 upregulated modulator of apoptosis
SDS: sodium dodecyl sulfate
TBST: tris‐buffered saline with 0.1% Tween 20 Detergent

## 1 Introduction

Ferroptosis is a form of regulated cell death that is characterized by the accumulation of lipid peroxides, which are toxic to cells and can lead to cell membrane damage [1]. The process involves the dysregulation of cellular iron homeostasis, leading to an increase in intracellular iron levels; this excess iron can then react with oxygen and lipid molecules in a process known as lipid peroxidation, generating reactive oxygen species (ROS) that damage cell membranes and organelles [2, 3, 4, 5]. Since ferroptosis is dependent on iron, targeting iron metabolism in cancer cells can induce ferroptosis [6, 7, 8]. This is achieved either through the use of iron chelators or inhibitors of iron uptake and storage proteins, or by inhibiting key antioxidant enzymes in cancer cells [9, 10, 11]. Combining ferroptosis‐inducing agents with other targeted anticancer therapies can also reportedly enhance the efficacy of treatment by targeting multiple pathways and overcoming resistance mechanisms in cancer cells [12, 13, 14]. A growing number of reports in this direction indicates that targeting ferroptosis in cancer cells represents a promising approach for the development of novel anticancer therapies.

Since its discovery in 1995, TRAIL has sparked growing interest among oncologists due to its remarkable ability to induce apoptosis in malignant human cells, but not in most normal cells [15]. In addition, we previously observed that ferroptotic agents such as erastin, artesunate, and sorafenib synergistically interact with apoptotic agent TRAIL and enhance TRAIL‐induced apoptosis [16, 17, 18, 19]. Data from our immunoblotting and microarray assays revealed that artesunate induces endoplasmic reticulum (ER) stress response through an increase in the level of unfolding proteins and promotes ATF4 (activating transcription factor 4)‐dependent *TRB3*, *ASNS*, and *CHOP* gene expression [16]. It is well known that the ER stress response mediated by the PERK‐eIF2α‐ATF4 pathway is involved in the regulation of expression of several target genes such as *CHOP* (C/EBP homologous protein) [20]. Our previous studies and literatures revealed that CHOP regulates PUMA (p53 upregulated modulator of apoptosis) and DR5 (death receptor 5) [16, 17, 21, 22]. The ER stress response plays an important role in the enhancement of TRAIL‐induced apoptosis [16, 23].

Artemisinins are a class of compounds, namely artemisinin (ART), artesunate (AS), arteether (AE), artemether (AM) and dihydroartemisinin (DHA), derived from the medicinal herb named *Artemisia annua* in China. While discovered originally for their efficacy against the malarial parasite, these drugs have demonstrated tumoricidal cytotoxicity against a variety of cancer cell lines, including breast, colon, and leukemia [16, 17, 24, 25]. Several such studies converge at the mechanistic understanding that iron (from Hb or free ions) can activate the endoperoxide bridge within these compounds [26, 27, 28, 29]. These compounds can trigger ferroptosis in cancer cells by generating reactive oxygen species (ROS) which results in lipid peroxidation and ER stress response [30, 31]. In this study, we hypothesized that the extent of the ER stress responses induced by artemisinin and its derivatives varies and is responsible for differential synergistic tumoricidal efficacy in TRAIL‐induced apoptosis. Our studies will provide information on which compound is the best for the clinical application of artemisinins in the combined therapy with TRAIL for cancer patients.

Our studies show that ER stress is a deterministic factor defining the death in cancer cells treated with a combination of artemisinins and TRAIL. We found this response to be dependent both on the artemisinins being used as well as the cell type used for treatment. It appears that DHA elicits the strongest cytotoxic response to the synergistic interaction with TRAIL compared to all the other derivatives used in this study. There are two major takeaways from this study: a) the minor differences in the chemistry of the various artemisinins could differentially sensitize cancer cells toward their apoptotic fate when the treatment is combined with TRAIL, and b) the ER stress response to artemisinins is a strong indicator of its potential to sensitize cancer cells to this synergistic apoptosis.

## 2 Materials and Method

### 2.1 Cell Lines and Culture Conditions

Human colorectal carcinoma line HCT116 and pancreatic cancer line BxPC3, both obtained from the American Type Culture Collection (ATCC, Manassas, VA). The HCT116 cells were maintained in McCoy's 5A medium, supplemented with 10% fetal bovine serum (FBS). The BxPC3 cells were maintained in high glucose DMEM medium, supplemented with 10% FBS and 1% Pen‐Strep (10,000 U, GIBCO). The cells were incubated in a humidified atmosphere of 5% CO_2_ at 37°C.

### 2.2 Chemical Reagents and Antibodies

Human recombinant TRAIL was produced as previously described [16]. AS, ART, and AE were purchased from Sigma‐Aldrich, and AM and DHA from Cayman Chemicals. All the artemisinins were obtained in powder form and stock solutions were prepared in sterile DMSO for treating cells in culture. The following primary antibodies were used for detection: anti‐ATF4 (catalog 11,815), anti‐CHOP (catalog 2895), anti‐caspase‐9 (catalog 7237), anti‐caspase‐8 (catalog 9746), anti‐DR5 (catalog 8074), anti‐poly (ADP‐ribose) polymerase (PARP) (catalog 9542), and anti‐rabbit IgG, HRP‐linked antibody (catalog 7074P2). Antibodies were purchased from Cell Signaling Technology (Beverly, MA, USA). Anti‐β‐actin (catalog A1978) was obtained from Sigma‐Aldrich. Goat anti‐mouse IgG‐HRP was purchased from Santa Cruz Biotechnology (Santa Cruz, CA).

### 2.3 Microscopy

Cells were grown on 35 mm Petri‐dishes and treated as usual. The adhered cells were viewed with appropriate lenses (10X and 40X objectives) compatible for phase‐contrast microscopy. The ECHO revolve microscope and its software was used for capturing images.

### 2.4 Cell Survival Assay

Cells were harvested and stained with 0.4% Trypan Blue solution. The Luna Automated Cell Counter (LB‐10001, Logos Biosystems, South Korea) was used to count live/dead cells. Stained cell suspension was loaded onto a counting slide compatible with the device and inserted into the counter to analyze cell count for each sample.

### 2.5 Immunoblotting and Densitometry Analyses

Cells were harvested and lysed in extraction buffer containing SDS (19.6% H_2_O, 24.9% Glycerol, 31.1% 0.5 M Tris, pH 6.8, 30% SDS, and 2.2%, 1% Bromophenol Blue). Proteins are quantified using the Pierce BCA Protein Assay Kit (Thermo). Finally, β‐mercaptoethanol is added to 25 μg of proteins per sample, boiled at 95°C, and loaded into wells for SDS‐PAGE. Gels are run using the Bio‐Rad Mini‐Protean Electrophoresis system and transferred onto nitrocellulose membranes using the Bio‐Rad wet‐transfer apparatus at a constant voltage of 90 V for 2 h at 4°C. Membranes were blocked in 5% non‐fat dry milk (Bio‐Rad) for 2 h at room temperature and incubated overnight with the appropriate primary antibody re‐suspended in 5% milk in TBST (50 mM Tris–HCl, pH 7.5, 150 mM NaCl, Tween‐20) at 4°C. Following 3 × 15 min washes using TBST (with 1% Tween 20), the membrane was incubated for 2 h at room temperature with the appropriate secondary antibody re‐suspended in 5% non‐fat dry milk (or bovine serum albumin (BSA) for select antibodies) in TBST at room temperature, followed by 3 × 15 min washes. ECL reagents (Millipore, Cat#: WBKLS0500) were used for signal detection and images were captured on the Bio‐Rad MP Chemidoc system. ImageJ was used to analyze intensities of the blots for densitometric comparison. All western blots were performed independently and in triplicates for significance in densitometric analyses.

### 2.6 Annexin V‐FITC and Propidium Iodide (PI) Staining Analysis

Apoptosis rate was evaluated using Annexin V‐FITC/PI Apoptosis Detection Kit (HY‐K1073, MedChemExpress). After treatment, cells were washed once with phosphate buffered saline (PBS). Then, 200 μL of binding buffer, 5 μL of Annexin V‐FITC and 10 μL of PI staining solution were added and mixed slightly. Cells were incubated at room temperature for 30 min in the dark, counterstained with Hoechst (1:2000 in PBS) for 10 min, and immediately observed under an ECHO fluorescence microscope.

### 2.7 LD_50_ Analysis

The value of LD_50_ for a substance which is the dose required to kill half the treatment with artemisinins was calculated using cytotoxicity data from survival experiments. Survival rates for treatment of each drug at each dosage (1 μM, 10 μM, 50 μM) were plotted on a scatter plot, and then a line of best fit was determined. The linear trendline's equation was used to calculate the estimated dosage (μM) required for a 50% survival rate (LD_50_) in each drug treatment alone as well as combined with TRAIL.

### 2.8 Combination Index (CI) Analysis

CI analysis was performed using CompuSyn software (ComboSyn Inc., Paramus, NJ, USA). The extent of antagonism/synergism was determined based on CI values using cytotoxicity data from survival experiments. CI values above 1 suggest antagonism between the drugs, whereas CI values below 1 indicate synergy. CI values in the 0.9–1.10 range mainly indicate additive effects; those between 0.9–0.85 suggest slight synergy, those in the range of 0.7–0.3 indicate moderate synergy, and those less than 0.3 suggest strong synergy.

### 2.9 siRNA Transfection

siRNA was transfected into cells using Lipofectamine 3000 (Thermo Fisher Scientific) and the manufacturer's protocol. Chop siRNA (#sc‐35,437) was used (Santa Cruz Biotechnology, Santa Cruz, CA). Cells were treated with siRNA and lipofectamine for 24 h and then drug treatment was performed for another 24 h. Cells were then harvested and analyzed using immunoblotting.

### 2.10 Statistics

All data values are shown in Mean +/− SD. Statistics is done by one‐way analysis of variance (ANOVA) or Student's *t*‐test, using GraphPad prism 7. Significance is shown for *p* value less than 0.05, where * = *p* < 0.05, ** = *p* < 0.01, *** = *p* < 0.005 and **** = *p* < 0.001.

## 3 Results

### 3.1 Structural Differences Between the Different Artemisinin and Its Derivatives

**FIGURE 1:**
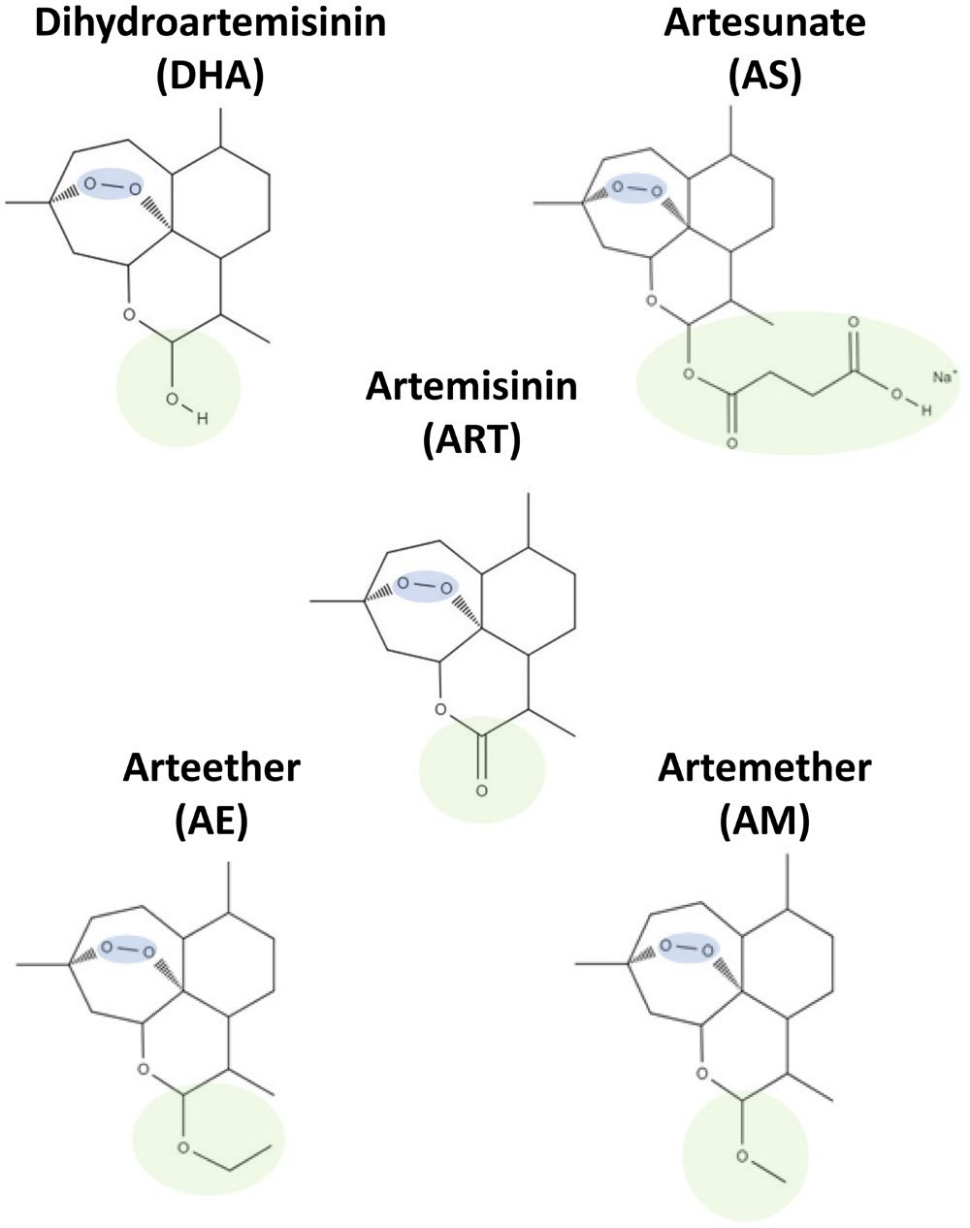
Schematic diagram of chemical structures of all the artemisinin and its derivatives. The chemical features of artemisinins, showing their similarity in the common endoperoxide bridge (highlighted in blue) and differences in the active groups bound to the C10 position (highlighted in green).

The five different artemisinins used for this study, namely artesunate (AS), artemisinin (ART), arteether (AE), artemether (AM), and dihydroartemisinin (DHA) share the same background. They have an endoperoxide bridge within a tetracyclic sesquiterpene scaffolding, highlighted in blue in all the chemical structures (Figure 1). Iron‐dependent activation of this peroxide is a requisite for the antimalarial activity shown by all members of the artemisinin family. The originally discovered compound, ART, is structurally a lactone. The functional moiety at its 2‐keto position is the functional group that gets modified for each compound. The C10 in the basic lactone ring shown undergoes hydroreduction to yield a lactol in DHA, whereas in AS, it is a four‐carbon ester group. For AE and AM, it is modified to ether and methyl ether groups, respectively (Figure 1). These modifications in the chemical structures within the members are important as they determine their specific formulations and methods of intake, as well as the potency of each drug. The structural modifications were introduced to increase the solubility and bioavailability of the compounds.

### 3.2 Different Artemisinins Induce Different Cytotoxicity

**FIGURE 2:**
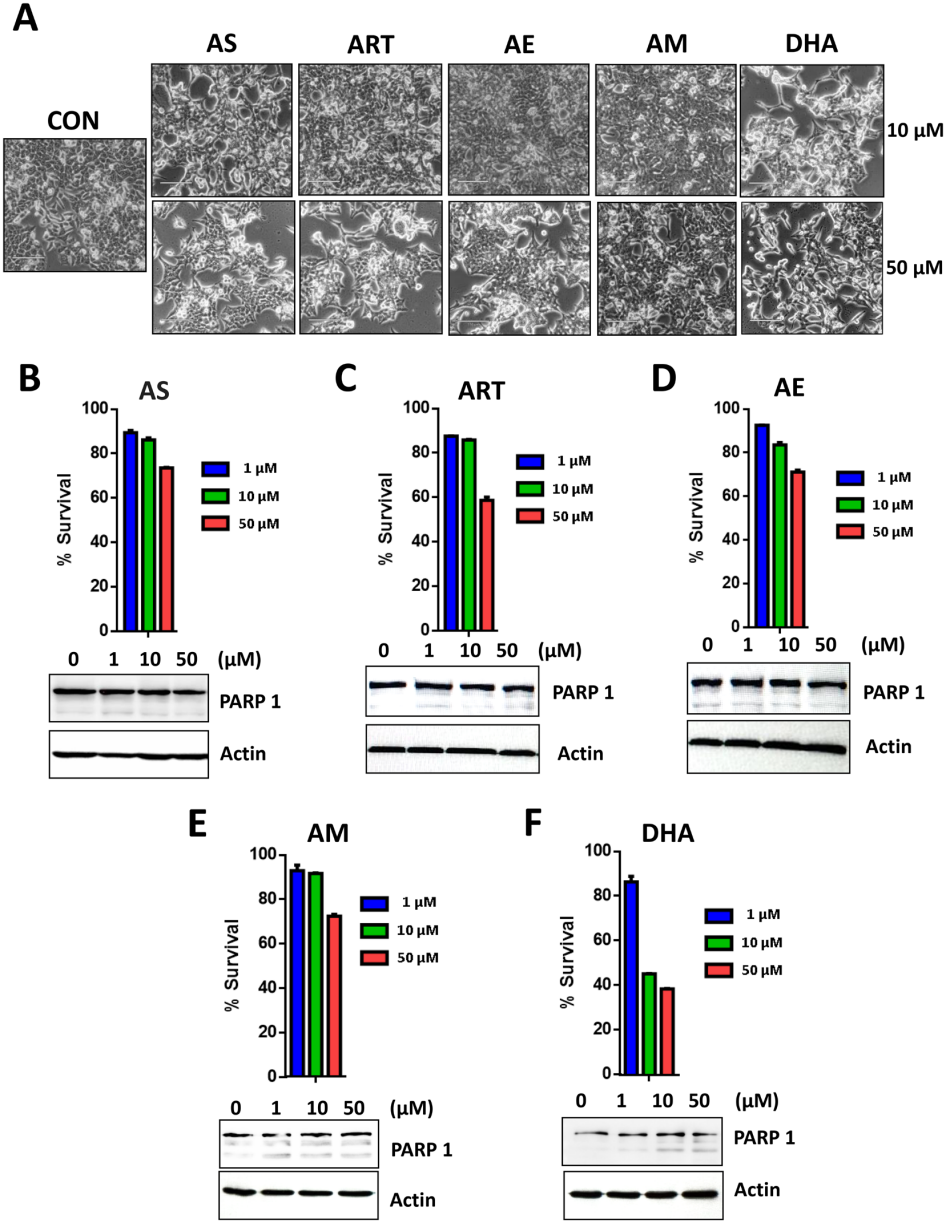
Assessment of artemisinin derivatives‐induced cytotoxicity. HCT116 cells were treated with various concentrations (1–50 μM) of artesunate (AS), artemisinin (ART), arteether (AE), artemether (AM), or dihydroartemisinin (DHA) for 24 h. (A) Phase‐contrast microscopy images were visualized under a light microscope. CON, 1% DMSO treated control cells. (B–F) Cell death was determined using trypan blue exclusion assay. Error bars represent mean +/− SD from triplicate experiments. Cell lysates were analyzed with immunoblotting assay using indicated antibodies. Actin was used as a protein loading control in each lane.

To examine the differential effect of artemisinin and its derivatives on TRAIL‐induced apoptosis, we first compared the cytotoxic effects of each drug. We treated HCT116 cells with all five drugs, and then assessed changes in cell survival. Treatment with various concentrations of each compound shows some toxicity, as depicted with phase‐contrast microscopy images in (Figure 2A). Cells were rounded up, detached, and appeared stressed due to treatment with artemisinin and its derivatives. Although cell growth was affected with 50 μM for all drugs, DHA showed significant toxicity. Counting live cells from the treatment, we also determined survival using the trypan blue exclusion assay and recorded the dose‐dependent cytotoxic effect for each derivative (Figure 2B–F). Table 1 shows that the value of LD_50_ for a substance, which is the dose required to kill half the treatment with artemisinins, was calculated using cytotoxicity data from survival experiments. Interestingly, the toxicity was maximum with DHA, where even with 10 μM treatment, the cell survival was halved (Figure 2F). Data from immunoblotting with the PARP‐1 antibody show that no prominent PARP‐1 cleavage occurred during treatment with artemisinins (Figure 2B–F). These results suggest that the cell death observed was not apoptotic in nature.

**TABLE 1:** LD_50_ calculations.

Drug	LD_50_	Unit
AS	74.155	μM
AS + TRAIL	49.822	μM
ART	58.515	μM
ART + TRAIL	32.112	μM
AE	69.089	μM
AE + TRAIL	27.902	μM
AM	70.999	μM
AM + TRAIL	66.114	μM
DHA	32.949	μM
DHA + TRAIL	3.461	μM

### 3.3 Synergistic Apoptosis Is Induced by Artemisinins in Combination With TRAIL

**FIGURE 3:**
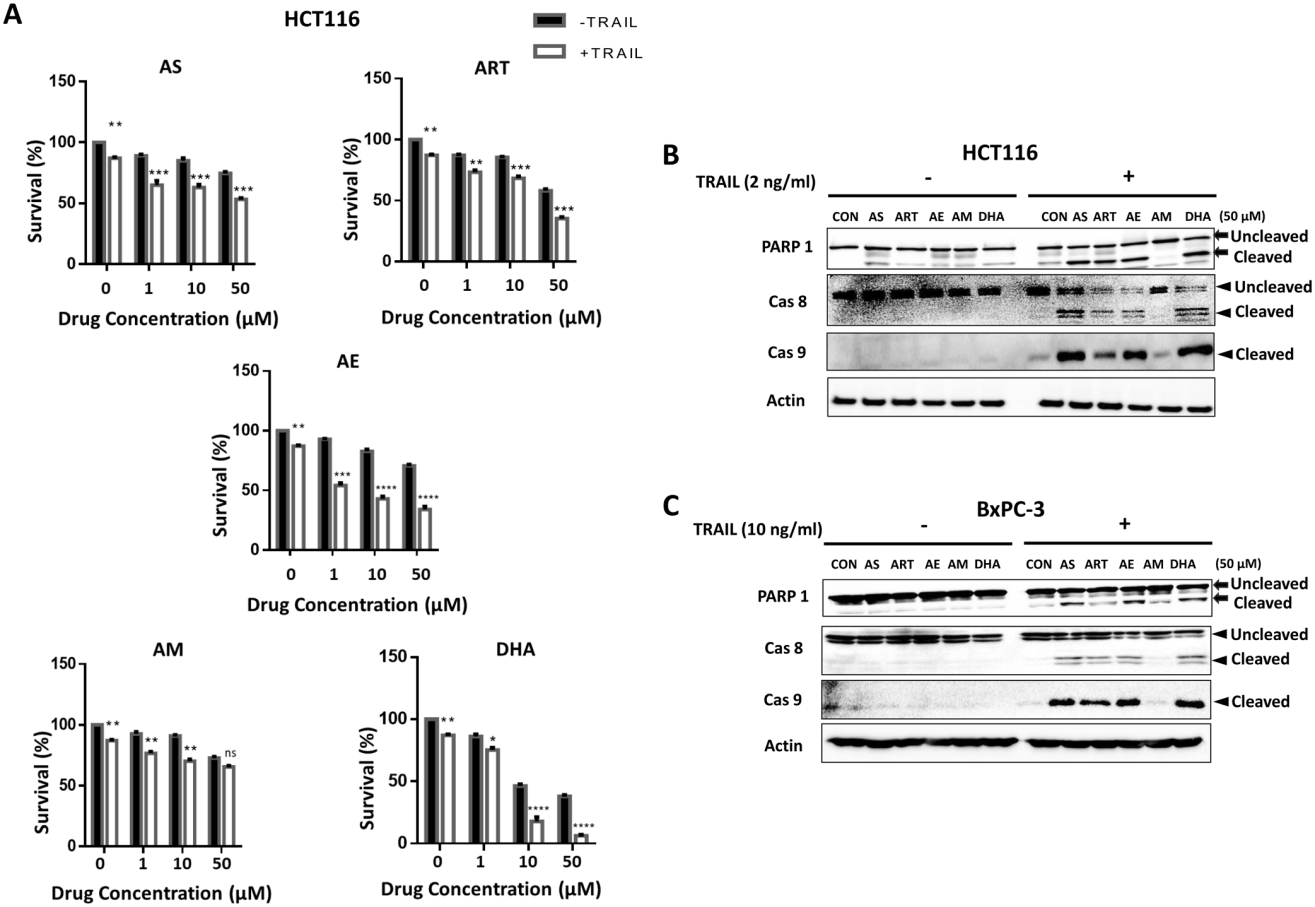
Effect of artemisinins on TRAIL‐induced apoptosis. HCT116 (A, B) and BxPC‐3 (C) cells were pretreated with artemisinins (1–50 μM) for 20 h and then treated without (−) or with (+) TRAIL (2 ng/mL for HCT116 cells or 10 ng/mL for BxPC‐3 cells) for an additional 4 h. (A) HCT116 cell survival was determined for dose‐dependent treatment with each of the drug with and without TRAIL. Error bars represent mean +/− SD from triplicate experiments. For statistical analysis, Student's *t*‐test (two‐sided, paired) was used. *p* values: *, 0.05; **, 0.01; ***, 0.005 and ****, 0.0001. (B, C) Cell lysates were analyzed with immunoblotting assay using indicated antibodies. Actin was used as a protein loading control in each lane.

**FIGURE 4:**
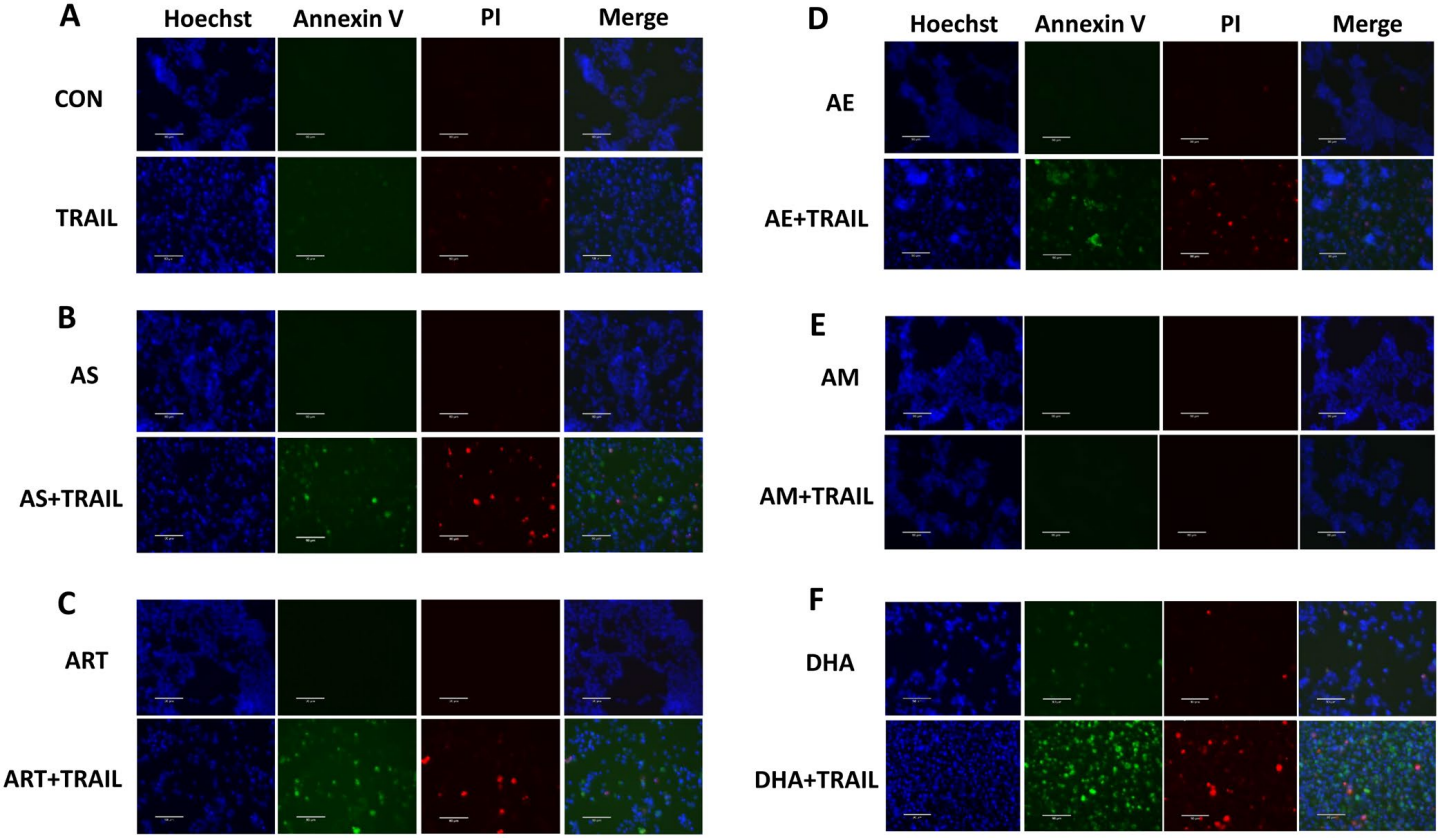
Effect of artemisinins on TRAIL‐induced apoptosis. HCT116 cells were pretreated with 50 μM artemisinins (A, control; B, AS; C, ART; D, AE; E, AM; F, DHA) for 20 h and then treated without or with TRAIL (2 ng/mL) for an additional 4 h. Cells were stained with Annexin V‐FITC (green), PI (red), and Hoechst (blue), and analyzed using an ECHO fluorescence microscope. Representative images are shown (scale bar: 100 μm).

In a previous study, we have shown that AS treatment in combination with TRAIL induces synergistic activation of apoptosis in cancer cells [16]. We investigated the synergistic activation of apoptosis during combined treatment of artemisinins and TRAIL. Cytotoxicity was markedly enhanced when the treatment of the derivatives, all except AM, was combined with TRAIL (Figure 3A). The extent of antagonism/synergism was also determined based on CI values using cytotoxicity data from survival experiments (Table 2). All the drugs except AM showed a strong synergy during combined treatment of artemisinins and TRAIL. We further examined whether promotion of cytotoxicity was due to an increase in apoptosis. As shown by immunoblotting for cleaved PARP‐1 and caspases 8 and 9 (a biochemical hallmark feature of apoptosis), the increase in cell death was due to apoptosis in both cell lines, HCT116 and BxPC3 (Figure 3B,C). All the drugs except AM seem to promote caspase activation and enhanced PARP‐1 cleavage. However, the extent of cell death obviously varied depending on both the compound as well as the cell type. In addition, Annexin V‐FITC/PI staining was performed for confirmation of apoptosis during treatment with artemisinins and TRAIL. Figure 4 shows that all the drugs except AM promoted apoptosis during treatment with artemisinins and TRAIL (Figure 4).

**TABLE 2:** CI index—synergism calculations.

10 ng/mL rhTRAIL +	CI value	Combined cytotoxic effect
1 μM Artesunate	0.00276	Strong Synergism
10 μM Artesunate	0.01886	Strong Synergism
50 μM Artesunate	0.00805	Strong Synergism
1 μM Artemisinin	0.44640	Moderate Synergism
10 μM Artemisinin	0.27722	Strong Synergism
50 μM Artemisinin	0.03430	Strong Synergism
1 μM Arteether	0.00293	Strong Synergism
10 μM Arteether	0.01036	Strong Synergism
50 μM Arteether	0.01346	Strong Synergism
1 μM Artemether	1.03675	Additive
10 μM Artemether	0.80969	Slight Synergism
50 μM Artemether	1.21326	Antagonism
1 μM Dihydroartemisinin	0.39044	Moderate Synergism
10 μM Dihydroartemisinin	0.05476	Strong Synergism
50 μM Dihydroartemisinin	0.09200	Strong Synergism

### 3.4 Different Artemisinins Induce Different Extents of ER Stress

**FIGURE 5:**
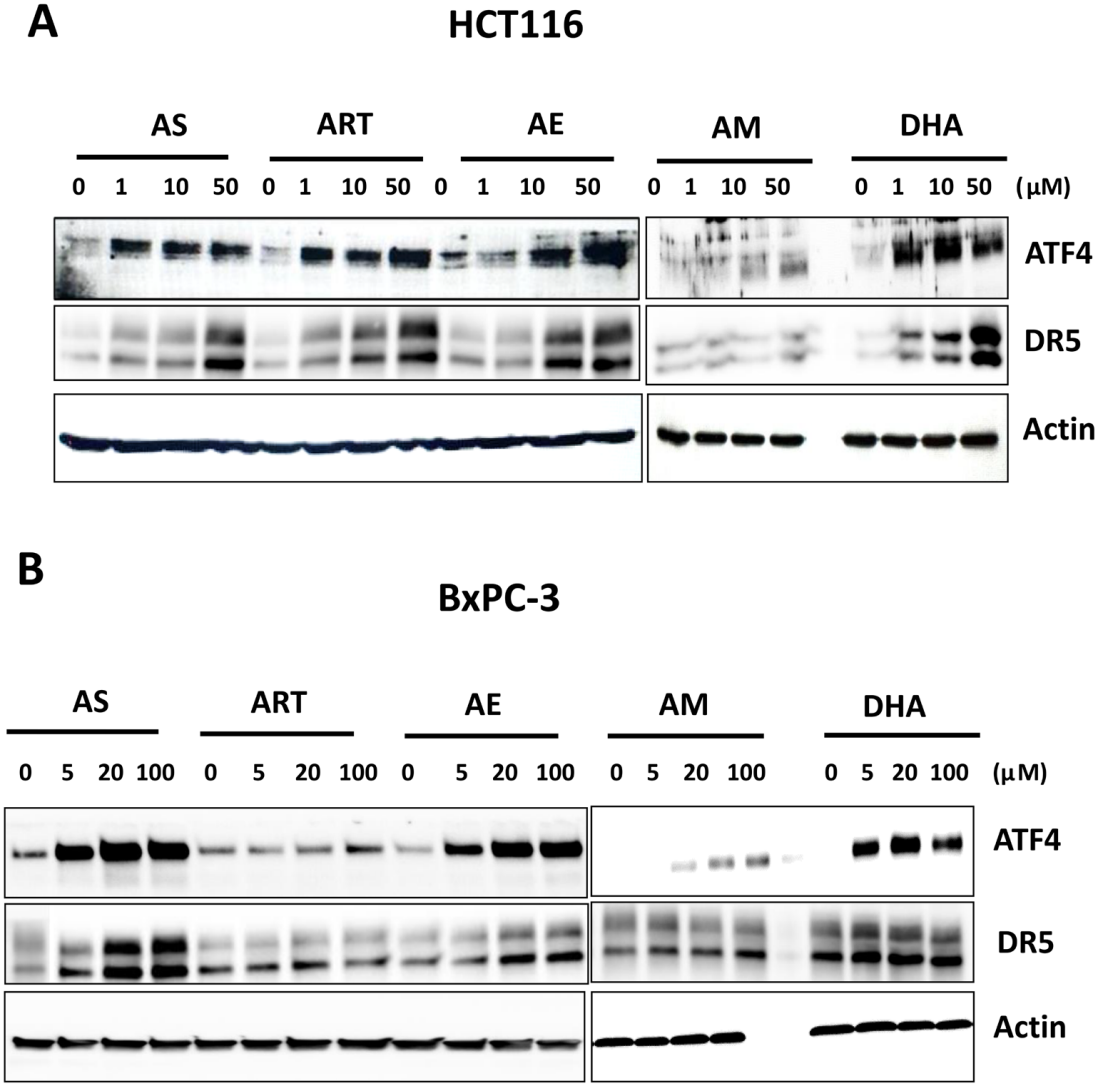
Assessment of ER stress response induced by artemisinins. HCT116 (A) and BxPC‐3 (B) cells were treated with various concentrations (1–100 μM) of artemisinin derivatives for 24 h. Whole‐cell lysates were analyzed using immunoblotting assay with indicated antibodies. Actin was used as a protein loading control in each lane.

We previously reported that combined treatment with AS and TRAIL does not enhance ferroptosis, characterized by an increase in lipid peroxidation [16]. Instead, AS causes ER stress, which is responsible for the induction of the synergistic apoptosis in response to combination treatment with AS and TRAIL [16]. Treatment with AS is shown to upregulate the expression of several ER stress‐related genes, including activating transcription factor 4 (ATF4) and TRAIL death receptor 5 (DR5) [16, 17]. To examine if the extent of ER stress from the different artemisinins would vary, we probed for ATF4 expression in cells treated with the range of the same concentrations of each compound. For HCT116, each drug was used to investigate a dose‐dependent response to treatments ranging from 0–50 μM. All compounds except AM induce ATF4 and DR5 expression in the HCT116 cells (Figure 5A). With BxPC3, which has a higher tolerance to artemisinins, we used a range of 0–100 μM (Figure 5B). Here too, we observed that the ATF4 expression was low with AM compared to the other drugs.

### 3.5 Synergistic Apoptosis Is a Function of Artemisinins‐Induced ER Stress Response

**FIGURE 6:**
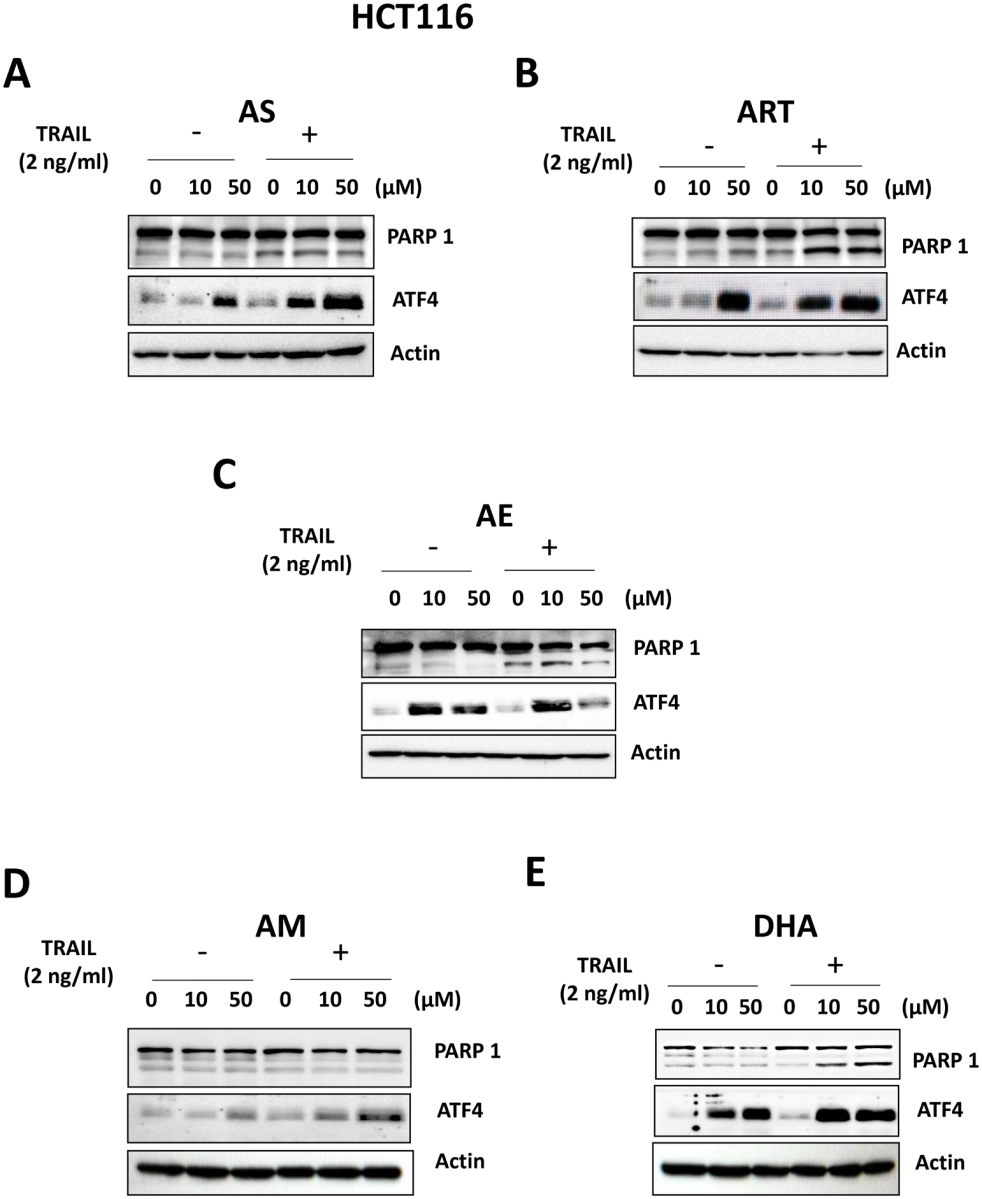
Comparison of ER stress response in artemisinin derivatives‐enhanced TRAIL‐induced apoptosis. (A–E) HCT116 cells were pretreated with various concentrations (10 or 50 μM) of artemisinins for 20 h and treated with or without 2 ng/mL TRAIL for an additional 4 h. PARP‐1 cleavage and ATF4 expression were analyzed by immunoblotting with respective antibodies for each drug.

The PARP‐1 cleavage and ATF4 expression level were examined in HCT116 cells treated with the same range of concentrations (0–50 μM) for each artemisinin and its derivatives. Data from immunoblotting show a dose‐dependent increase in cleaved PARP‐1 with increasing concentration of each compound except AM, indicating apoptotic death when treated in combination with TRAIL (Figure 6A–E). In the absence of TRAIL, however, the ATF4 expression did not result in apoptosis. This corroborates our earlier observation in both HCT116 and BxPC3 lines. The synergistic apoptosis observed, therefore, seems to be determined by the extent of ATF4 response each compound elicits even before TRAIL acts, but TRAIL is necessary for the apoptosis.

### 3.6 Role of CHOP in the Synergistic Effect of Artemisinins on TRAIL‐Induced Apoptosis

**FIGURE 7:**
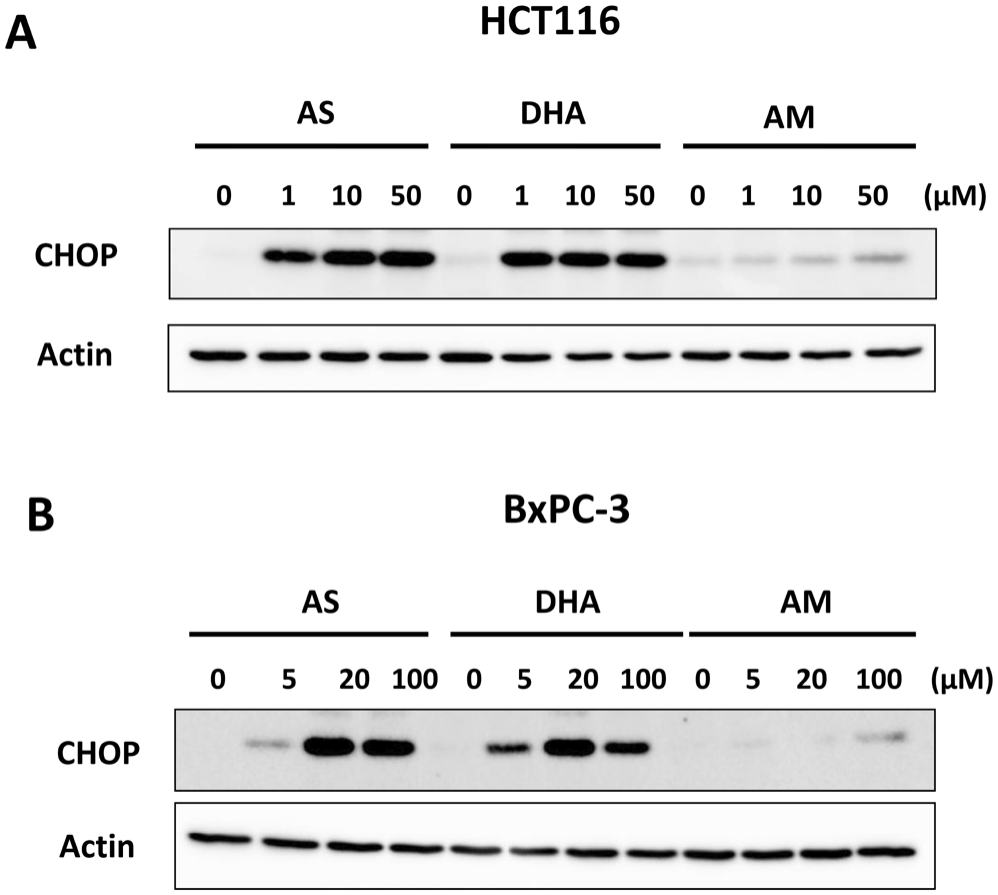
Comparison of ER stress response in AS, DHA, and AM. HCT116 (A) and BxPC‐3 (B) cells were treated with various concentrations (1–50 μM) of AS, DHA, or AM for 24 h. Whole‐cell lysates were analyzed using immunoblotting assay with indicated antibodies. Actin was used as a protein loading control in each lane.

**FIGURE 8:**
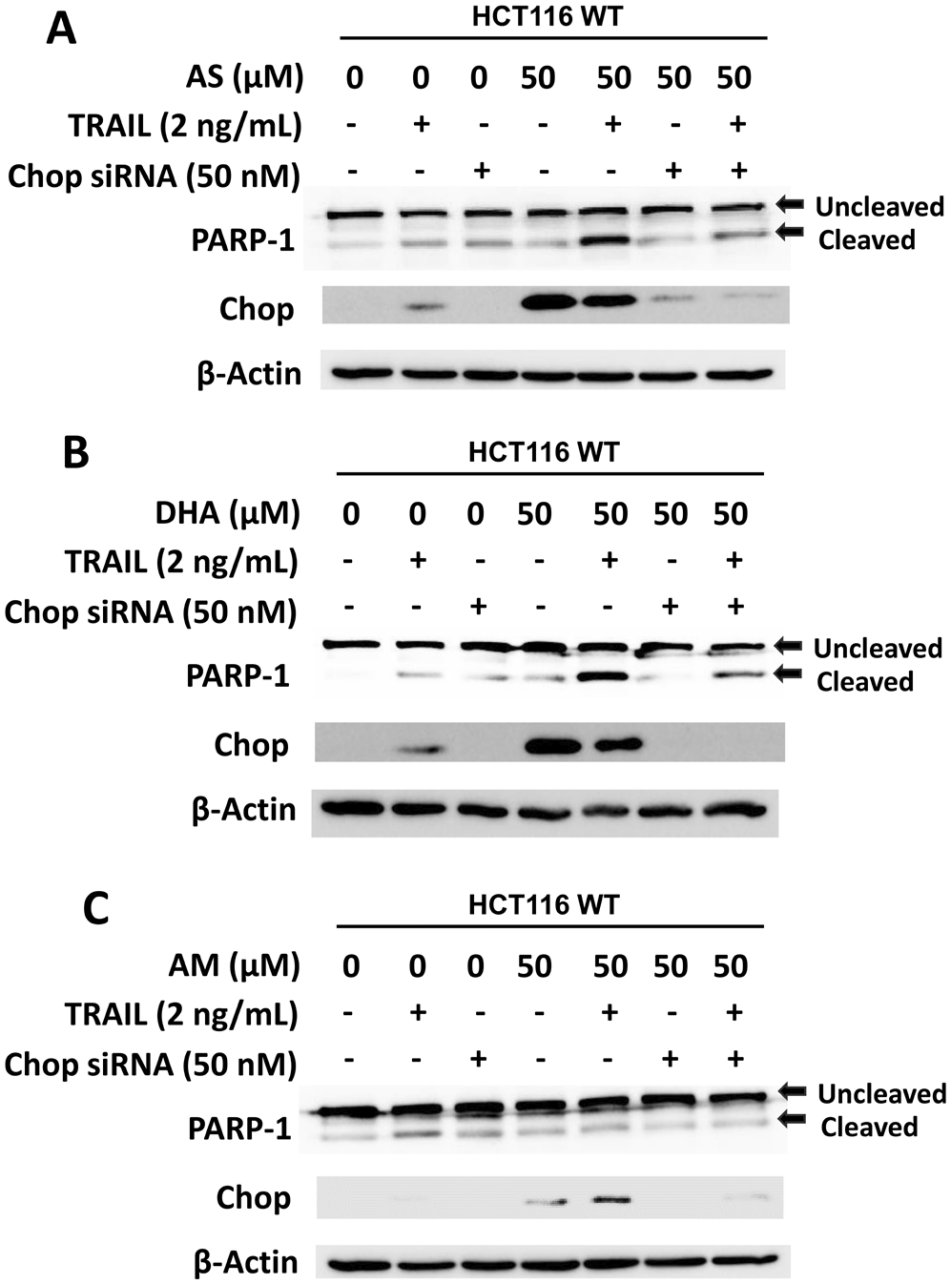
Role of CHOP in artemisinin derivatives‐enhanced TRAIL‐induced apoptosis. HCT116 cells were transfected with siRNA (50 nM) of CHOP for 24 h. After transfection, cells were pretreated with 50 μM artemisinins (A, AS; B, DHA; C, AM) for 20 h and then treated without or with TRAIL (2 ng/mL) for an additional 4 h. Whole‐cell lysates were analyzed using immunoblotting assay with indicated antibodies. Actin was used as a protein loading control in each lane.

We previously reported the involvement of ER stress response (the PERK‐eIF2α‐ATF4‐CHOP) pathway in the enhancement of TRAIL‐induced apoptosis [16, 32]. Our studies revealed that inhibition of the ER stress response (PERK‐eIF2α‐ATF4‐CHOP) pathway suppressed the enhancement of TRAIL‐induced apoptosis by ferroptotic agent treatment. The role of CHOP in the artemisinins‐enhanced TRAIL cytotoxicity. Firstly, we examined the *CHOP* gene expression during treatment with artemisinins. Figure 7 shows that AS and DHA except AM significantly enhanced the level of CHOP. Next, we investigated the role of CHOP in the synergistic effect of artemisinins on TRAIL‐induced apoptosis was using siRNA knockdown techniques. HCT116 cells were transfected with CHOP siRNA and then treated with AS, DHA, or AM followed by TRAIL. The successful transfection of CHOP siRNA was confirmed by western blotting results (Figure 8). Figure 8 shows that AS and DHA except AM enhanced TRAIL‐induced apoptosis and knockdown of *CHOP* gene expression significantly inhibited the promoting effect of AS and DHA. Our results confirmed the involvement of ER stress response in the artemisinins‐enhanced TRAIL‐induced apoptosis.

### 3.7 Analyzing the Relationship Between Enhancement of Apoptosis and ER Stress Response

**FIGURE 9:**
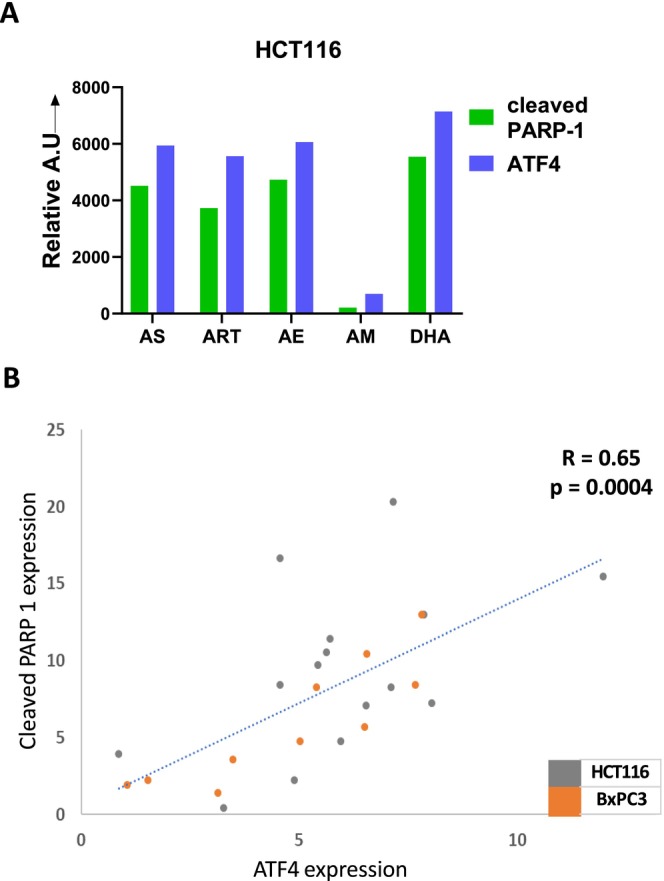
Correlation between PARP cleavage and ATF4 gene expression during treatment with artemisinins and TRAIL. Data from Figures 3, 5, and 6 are summarized. (A) Bar graphs showing the normalized expression levels of ATF4 and cleaved PARP‐1, highlighting their correlation in HCT116 cells treated with 50 μM artemisinin and 2 ng/mL TRAIL. (B) The expression of cleaved PARP and ATF4 for treatments over different concentrations with the five different artemisinins in HCT116 and BxPC‐3 cells is plotted; correlation coefficient, *R* and *p* value is indicated for analyses of experimental data in triplicates.

We analyzed the correlation between cleaved PARP‐1 and ATF4 expression with a combination treatment of the different artemisinins with TRAIL, in both HCT116 and BxPC3. The similarity in the expressional trend of these two proteins is charted in stacked area graphs, for treatment with 10 μM of each artemisinin with TRAIL, 2 ng/mL for HCT116 (Figure 9A) and 10 ng/mL for BxPC3 cells (Figure 9B). A correlation between PARP‐1 cleavage and ATF expression for all the treatments in HCT116 and BxPC‐3 cells was summarized in Figure 9B. Cleaved PARP and ATF4 expression, for treatments with all five different ART derivatives over three different doses, is positively correlated with a correlation coefficient (*R*) > 0.6 that is highly significant (*p* < 0.0005) (Figure 9C).

## 4 Discussion

Our results confirm that the synergistic activation of apoptosis in cancer cells, when treated with a combination of artemisinins and TRAIL, is a result of the ER stress response to the artemisinin derivatives. This also supports our hypothesis that not all the artemisinin derivatives are equal in efficiency. As shown in Figures 2 and 3, the extent of ER stress induction varied with artemisinins, and so did the killing efficiency when treated in combination with TRAIL. Cell survival decreased with dose‐dependent increases in the concentration of artemisinins used in combination with TRAIL to treat the cells. The correlation between ATF4 expression and cleaved PARP‐1 level was highly significant (Figure 9B), and this holds for all the artemisinins in both cell types.

Data from our current studies in Figures 5 and 7 and previous studies show that artemisinins, except AM, significantly induce the ER stress response. The ER stress induced by artemisinins is likely due to ROS generation, calcium imbalance, and direct damage to ER proteins. Artemisinins contain an endoperoxide bridge that, when activated in the presence of iron, may produce ROS through the Fenton reaction. These ROS can oxidize proteins and lipids in the ER, impairing protein folding and causing accumulation of misfolded proteins. Another possibility is that artemisinins may disturb ER calcium levels, which are critical for proper protein folding and chaperone function. Calcium efflux from the ER can contribute to ER stress and mitochondrial dysfunction. The other possibility is that artemisinins may covalently modify thiol‐containing proteins, including ER‐resident chaperones and enzymes like protein disulfide isomerase (PDI), disrupting their function. They may also impair post‐translational modifications, such as N‐linked glycosylation, causing misfolded glycoproteins to accumulate in the ER. Our previous studies and several researchers reported that artemisinins trigger unfolded protein response (UPR) signaling pathways such as the PERK‐eIF2α‐ATF4‐CHOP signal transduction pathway [16, 19, 32]. This signaling pathway plays an important role in the interaction between artemisinins and TRAIL. The combined treatment of TRAIL and artemisinins, except AM, promotes TRAIL‐induced apoptosis, and its promotion is suppressed in CHOP knockdown cells (Figure 8). These results suggest that the crosstalk between the ER stress response sensor‐associated signaling pathway and the TRAIL‐induced apoptotic signaling pathway orchestrates synergistic cytotoxicity during the combined treatment.

In the present study, we observed no synergistic interaction between AM and TRAIL. Although AM is structurally similar to other artemisinin derivatives, our studies show reduced or absent induction of ER stress compared to other compounds. Several possible explanations could account for this difference. AM is significantly more lipophilic than other derivatives due to its methyl ether group. This may alter its subcellular distribution, potentially reducing accumulation in the ER lumen. It may also cause it to preferentially partition into lipid membranes (e.g., mitochondria or plasma membrane) rather than the ER. Another possibility is that AM is a less potent ROS generator compared to other artemisinin derivatives. Since ROS are a key trigger of ER stress, lower ROS levels might explain the absence of ER stress induction. Another possibility is that unlike other artemisinin derivatives, AM may lack sufficient reactivity with ER‐resident proteins due to steric hindrance from its methyl ether group or lower chemical reactivity of its peroxide bridge in certain cellular contexts. The other possibility is that AM may be more metabolically stable and requires conversion to DHA for full activity. Due to low metabolic conversion capacity, AM may not produce sufficient active metabolites to cause ER stress. Furthermore, AM may not reach critical concentrations in the ER due to lower aqueous solubility and preferential sequestration into lipid droplets or non‐ER organelles. Obviously, further studies are necessary to understand the molecular mechanism of difference between AM‐induced ER stress and other artemisinin derivatives‐induced ER stress.

In this study, we observed that differential induction of ER stress response by treatment with artemisinin and its derivatives. We found DHA to be the best of all the drugs, in that it causes the maximum death with the same molar concentration (Figure 3A). Unlike DHA, the least response was seen to AM. These agents are known to have tumoricidal efficacy against multiple cancer types through various pathways [33, 34, 35]. Several researchers have revealed that activation of ER stress and generation of ROS may play an important role in the antitumoral activity [36, 37, 38]. Since oxidative stress is caused by an imbalance between oxidant production and antioxidant reserves [39], DHA may be the most effective agent to induce an imbalance between them. Previous studies reveal that DHA induces ROS production, causes mitochondrial damage, and activates autophagy via stimulation of the ROS/Erk1/2 pathway [40]. DHA also induces lysosomal superoxide production, leading lysosomal membrane permeabilization (LMP), and autophagic flux blockage [40]. In addition, DHA is the active metabolite of all artemisinin compounds and formulated in a direct oral preparation [41]. Here, we propose that a combination of DHA and TRAIL can be useful to treat colorectal cancer and pancreatic cancer patients. It might be worth noting though that not all artemisinins have the same bioavailability. While the higher solubility of DHA and AS in polar solvents result in restricted volume of distribution, which helps in targeting solid tumors effectively, the highly lipophilic AM and AE administered through intramuscular injections, have a better tissue distribution [42]. This adds another layer of complexity to the interpretations of our observations drawn in simplistic experimental models, with cell lines treated with all drugs solubilized in DMSO. Realistically, multiple parameters like the chemical formulation and mode of administration decide the pharmacokinetics of a drug in patients.

The repurposing of artemisinins for cancer is still relatively new and additional studies into the mechanisms of augmenting cell death can add to the understanding of how the different artemisinin and its derivatives compare, thereby promising an attractive anticancer therapy. In addition to being generally well tolerated in humans, artemisinins are also widely accepted as first‐line treatments for malaria, which would ease the process of clinical development and regulatory approval for their use in cancer treatment [33, 34, 35]. The importance of the ER response to ferroptotic agents, and its role in sensitizing cancer cells for TRAIL therapy is worth exploring more, especially in terms of identifying the best drug available. More studies using animal models and clinical trials will help establish dosage in patients, for the use of artemisinins, particularly DHA in combination with TRAIL for treating solid cancers.

However, regarding the potential synergistic use of artemisinin derivatives together with TRAIL in cancer treatment, two major limitations can be envisaged. First, the short half‐life of artemisinins may not be useful for long‐term treatment of cancer diseases. To overcome this obstacle, we will develop heptamethine carbocyanine DZ‐1 dye conjugated to DHA (DZ‐1‐DHA). Researchers have reported that near‐infrared heptamethine carbocyanine dye can enter cancer cells with high selectivity via the organic anion transporting polypeptide family of carrier proteins, which are differentially expressed in cancer cells [43, 44, 45]. DZ‐1‐DHA would preferentially accumulate in tumor tissue [43, 44, 45, 46]. Second, the clinical use of TRAIL has shown strong limitations due to poor pharmacokinetics, short half‐life, and rapid onset of resistance [47, 48]. To overcome these limitations, we have developed a secretory TRAIL‐armed natural killer (NK) cell‐based therapy [49]. We previously reported that secretory TRAIL‐armed NK cells are able to accumulate selectively at tumor sites and exert tumoricidal effects through sustained TRAIL release [49]. In addition, researchers have developed an immunoglobulin Fc domain‐fused TRAIL (Fc‐TRAIL) [50, 51]. The chimeric fusion protein containing the fragment crystallizable region (Fc) of human immunoglobulin increases the plasma half‐life of the fusion protein and improves apoptotic activity [50, 51, 52, 53]. More importantly, no short‐term toxicity, especially liver toxicity, was observed [50]. Thus, the development of a combined treatment with DZ‐1‐DHA and secretory TRAIL‐armed NK cells/chimeric Fc‐TRAIL needs to be carried out in the near future.

## Author Contributions

**Yong J. Lee:** conceptualization (lead), funding acquisition (lead), project administration (lead), writing – review and editing (lead). **Snigdha Bhowmick:** data curation (lead), investigation (lead), methodology (lead), writing – original draft (lead).

## Conflicts of Interest

The authors declare no conflicts of interest.

## Supporting information


**Data S1.** Supporting Information.

## Data Availability

Data and materials will be made freely available to the scientific research community as soon as they have been documented in a publication.
