# Supplementary material for: ER Stress Induced by Artemisinin and Its Derivatives Determines the Susceptibility to Their Synergistic Apoptotic Killing With TRAIL
Source: Cancer Med. 2025 Jun 23;14(12):e71001. doi: 10.1002/cam4.71001 (PMC12183610; doi:10.1002/cam4.71001)
Supplement: Supplementary file 1 — Data S1. Supporting Information. [file CAM4-14-e71001-s001.pdf]

Figure 2

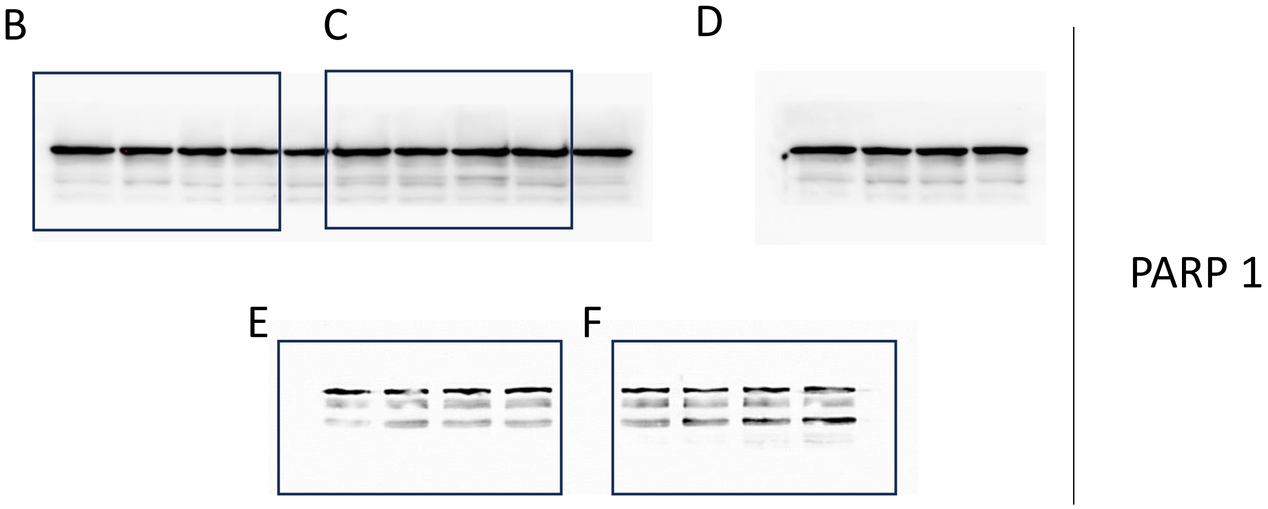

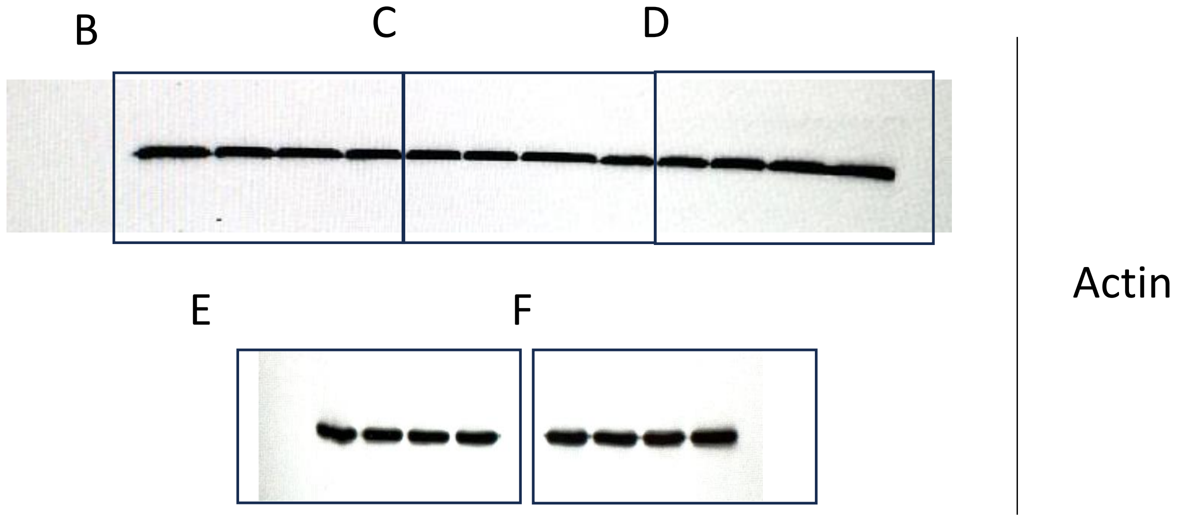

Figure 3

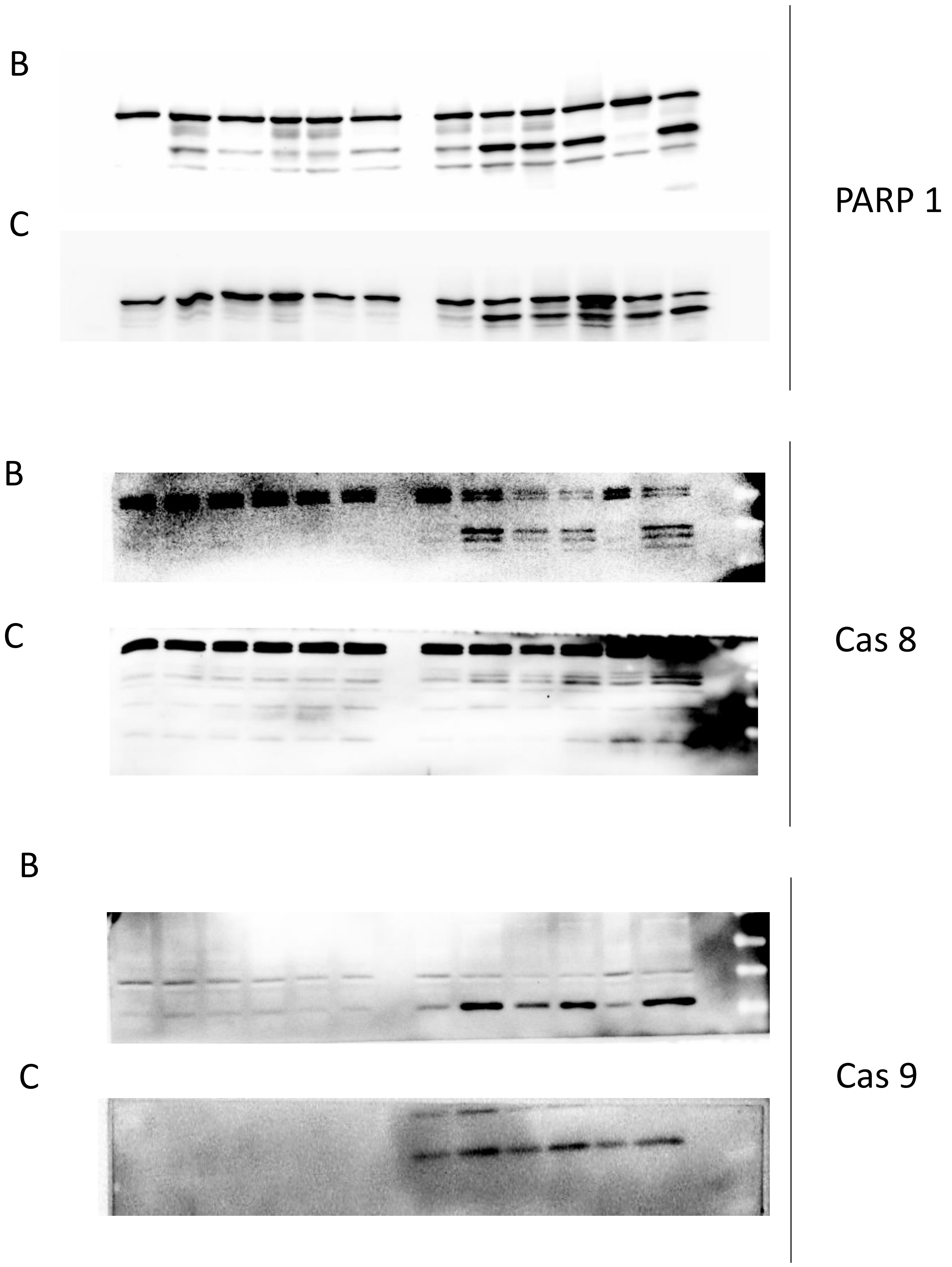

Actin blots on P5

Figure 4

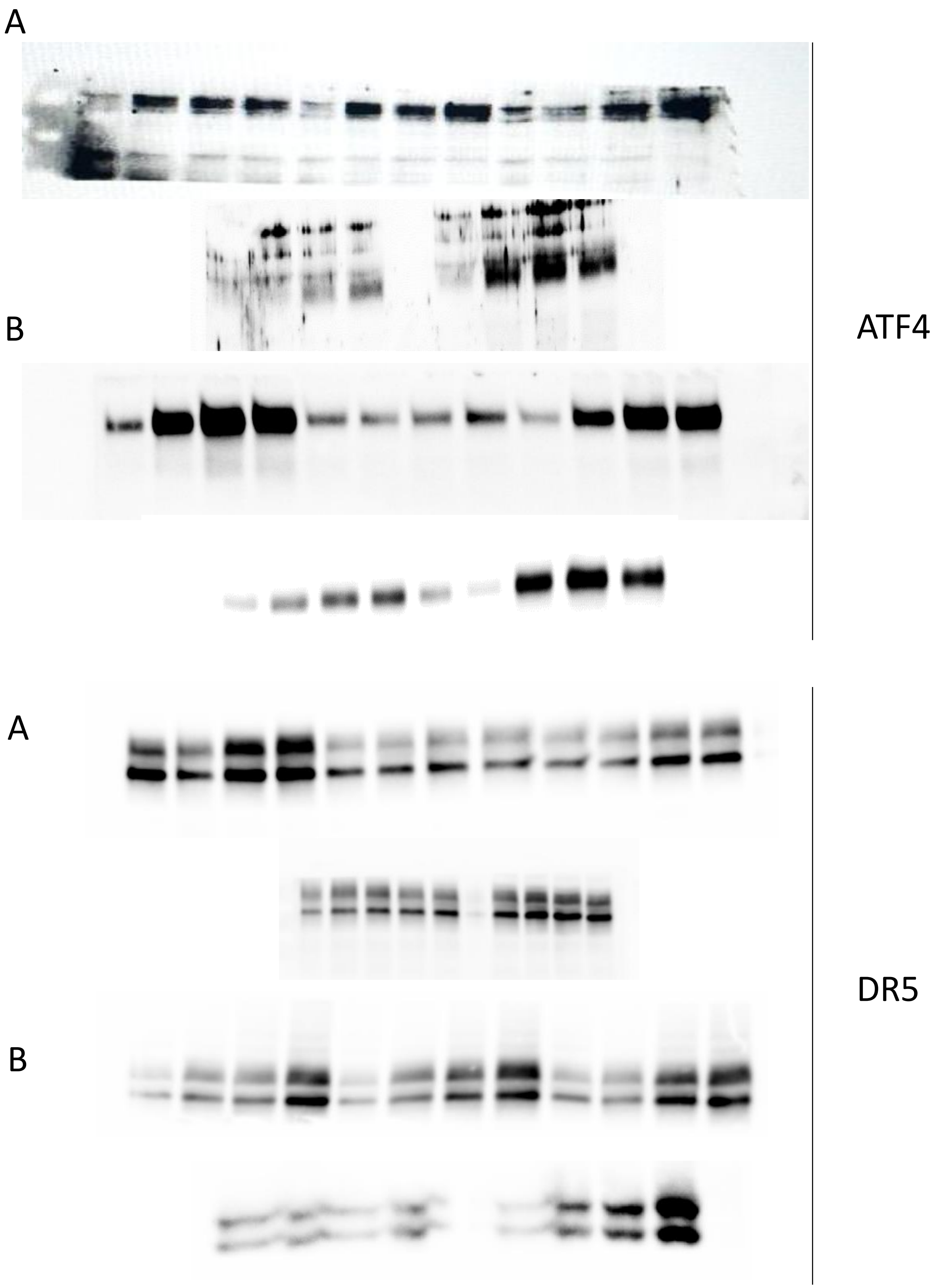

Actin blots on P5

Figure 5

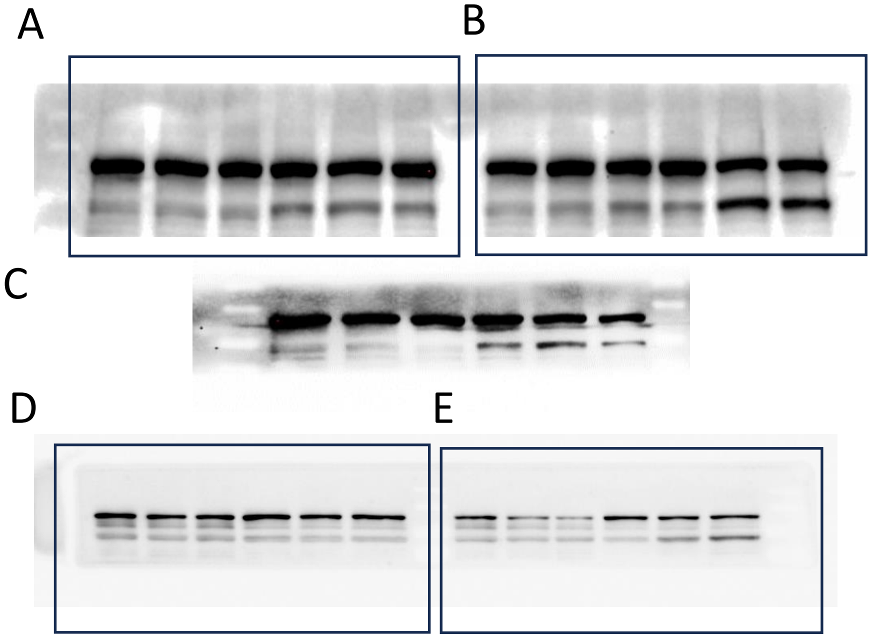

PARP 1

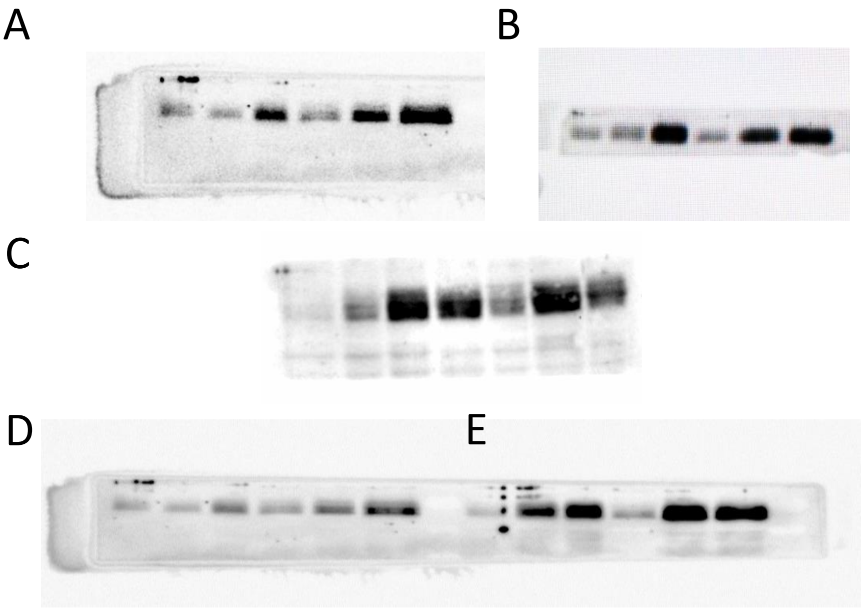

ATF4

Actin blots on P5

Actin Blots

A

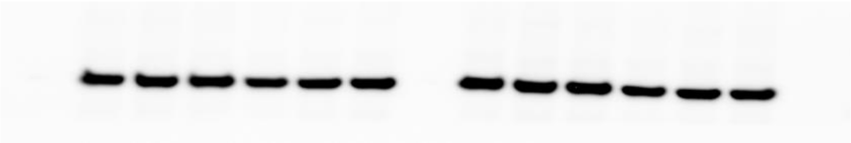

B

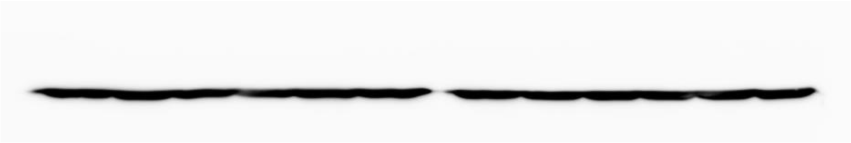

Figure 3

A

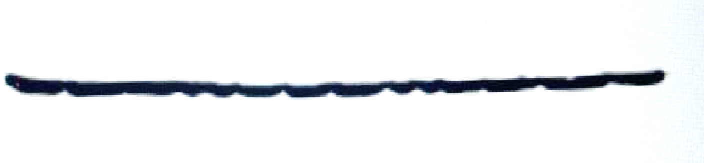

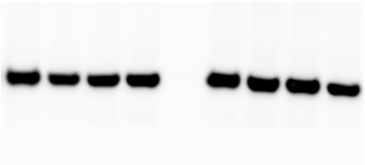

B

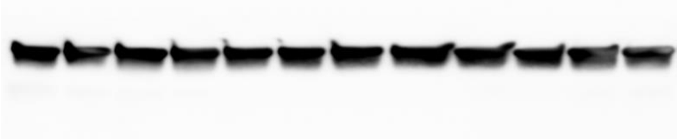

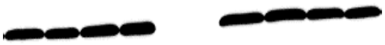

Figure 4

A B

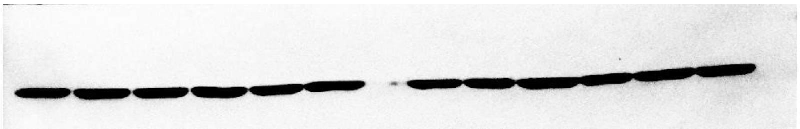

C

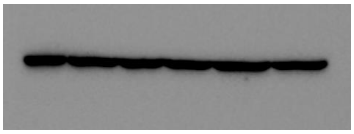

D E

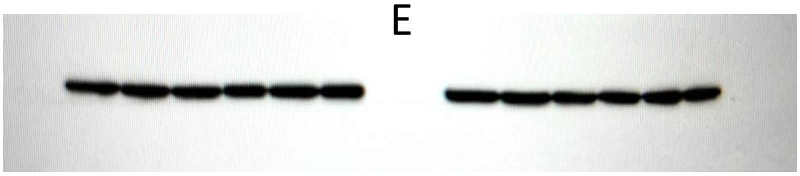

Figure 5
